# iTRAQ-Based Proteomic Analysis Reveals Molecular Pathways in Potato Cold Stress Response

**DOI:** 10.3390/life15060885

**Published:** 2025-05-30

**Authors:** Huiju Yang, Mingwei Chen, Huachun Guo

**Affiliations:** 1 Lijiang Normal University, Lijiang 674199, China; 2 College of Agronomy and Biotechnology, Yunnan Agricultural University, Kunming 650205, China

**Keywords:** cold stress, differentially abundant proteins (DAPs), isobaric tags for relative and absolute quantification (iTRAQ), pathways, potato

## Abstract

Potato (*Solanum tuberosum* L.) is an essential crop for food and industrial production, but its cultivation in low-temperature regions is challenging. Therefore, using proteomics quantification technology based on isobaric tags for relative and absolute quantification, we investigated molecular responses in potato leaves under cold stress at 1 (L1), 3 (L3), and 6 h (L6), with normal growth as a control. A total of 3292 proteins at all stages were identified, with 125, 250, and 380 differentially abundant proteins at L1, L3, and L6, respectively. The GO and KEGG analysis revealed that these DAPs were linked to photosynthesis, oxidative phosphorylation, and the biosynthesis of ansamycins. Further association analysis showed down-regulation in aminoacyl-tRNA biosynthesis and up-regulation in pathways like alpha-linolenic acid metabolism. At L6, a significant up-regulation of cold tolerance genes was observed. This study provides insight into the proteomic mechanisms of cold tolerance in potato, laying a foundation for genetic breeding.

## 1. Introduction

Biotic and abiotic stress influence plants during the growth process, causing changes in the morphological, physiological, biochemical, and molecular levels of plants, thus they might pose adverse effects on plant growth and crop output [1,2,3,4,5].

Potato is a root vegetable that is native to the Americas and is a member of the Solanaceae family [6,7]. While exhibiting tolerance to various environmental stresses, potatoes are particularly sensitive to hypothermia, which often leads to great yield losses due to cold damage [8]. Given the importance of potato in several aspects, it is essential to investigate the molecular mechanism underlying the cold resistance and enhance this trait through modern molecular breeding methods to improve both productivity and resistance.

The transcriptional and metabolic network is a cold adaptation mechanism in plants [6,9,10,11,12]. Several studies have demonstrated that a full genome transcript analysis, along with mutational and transgenic plant analysis, is valuable in investigating the complex transcriptional system involved in cold acclimation in plants. Rihan et al. conducted transcriptome profiling of approximately 8000 genes of *Arabidopsis*, uncovering several regulatory pathways involved in the cold response. Of the 300 genes being affected by cold, 218 exhibited an increase in transcript expression, while 88 showed a decrease after a 7-day cold treatment [13].

Among the genes above, the *CBF* gene plays pivotal role in low-temperature adaptation and signal transduction, and has been identified in a wide range of plants [14]. Numerous studies have demonstrated the essential role of the CBF/DREB1 transcription factor family in cold acclimation and freezing stress tolerance in *Arabidopsis* plants [15,16]. These transcription factors bind to specific regulatory sequences in the promoters of cold- and dehydration-response genes, and the regulatory sequences are C-repeat (CRT: TGGCCGAC) and the dehydration response element (DRE: TACCGACAT) [17]. Both sequences contain a highly conserved core 5 bp sequence of CCGAC, which regulates transcription in response to drought, low temperature, and salinity [18,19]. The CBF protein is a DNA binding protein that recognizes the DNA regulatory elements CRT/DRE located in the cold-regulated (*COR*) gene promoter, thereby controlling the induction of *COR* gene expression [20].

While progress has been made in studies on the plant transcriptome network of low-temperature tolerance, the protein changes and regulation encoded by these gene locks remain poorly understood. Given the crucial role of proteins in nearly all cellular functions, the application of proteomics has emerged as a critical technique for gaining deeper insight into the biological mechanisms [21,22,23]. Proteomic analysis enables the comprehensive qualitative and quantitative profiling of proteins. In particular, iTRAQ technology allows for multiplexed quantification, making it effective in elucidating the functional roles of specific proteins [24,25,26,27]. Therefore, in this study, we used iTRAQ-based protein quantification technology to investigate potato proteome changes and responses under low-temperature stress.

## 2. Materials and Methods

### 2.1. Plant Materials and Experimental Design

To explore the cold stress response mechanism of potato at the protein level, the potato (*Solanum tuberosum* L.) commercial cultivar ‘Lishu 6’ (L), which has a strong cold-tolerance, was used for all experiments. The seeds of a potato cultivar were sown in small plastic pots (12 cm in diameter × 12 cm in height) containing a commercial mixture of soil/peat/compost (50:30:20, *v*/*v*/*v*) and grown in a temperature-controlled greenhouse (20 °C/18 °C, day/night) under a natural photoperiod. The plants were regularly watered.

For low-temperature treatment, 35-day-old uniformly grown seedlings were exposed to a −2 °C environment. Leaf samples were collected at 0 h (NT), 1 h (L1), 3 h (L3), and 6 h (L6) following the onset of cold exposure. The NT group served as the untreated control. At each time point, six leaves were collected and pooled per sample. There were six biological replicate samples in each group, so a total of 24 samples were used in the HPLC-MS/MS and transcriptome analyses. These collected samples were frozen in liquid nitrogen and stored at −80 °C. Prior to extraction, the samples were thoroughly ground into a fine powder.

### 2.2. Protein Digestion

Protein digestion was performed using an in-solution tryptic digestion protocol as described by Lan et al. [28]. Potato proteins were precipitated using the ProteoExtract Protein Precipitation Kit (EMD Biosciences, Darmstadt, Germany), resuspended in 500 mM triethylammonium bicarbonate (TEAB) and quantified using the bicinchoninic acid (BCA) assay (Beyotime, Beijing, China) [29]. A total of 100 μg of protein were used for digestion. After digestion, an equal volume of 0.1% formic acid (FA) was added to acidify the sample. Peptides were purified using a Strata-X C18 column (Phenomenex, Torrance, CA, USA), pre-activated with methanol and equilibrated three times with 1 mL of 0.1% FA. The column was then washed twice with 0.1% FA containing 5% acetonitrile (ACN), and peptides were eluted with 1 mL of 0.1% FA containing 80% ACN. The eluted peptides were dried using a vacuum concentrator and reconstituted in 20 μL of 0.5 M TEAB for labeling. The peptides were labeled using the iTRAQ 8-plex reagent (Sciex, Framingham, MA, USA) according to the manufacturer’s instructions, pooled, and lyophilized using a vacuum concentrator.

### 2.3. High Performance Liquid Chromatography-Tandem Mass Spectrometry (HPLC-MS/MS) Analysis

An LC-ESI-MS/MS analysis was performed using an AB SCIEX nanoLC-MS/MS (Triple TOF 5600 plus) system (AB SCIEX Company, Framingham, MA, USA). The samples were chromatographed using a 90-min gradient of 2–30% (buffer A 0.1% (*v*/*v*) formic acid, 5% (*v*/*v*) acetonitrile, buffer B 0.1% (*v*/*v*) formic acid in deionized water, 95% (*v*/*v*) acetonitrile) after direct injection onto a 20 μm PicoFrit emitter (New Objective, Boston, MA, USA) packed to 12 cm with a Magic C18 AQ 3 μm 120 Å stationary phase. The MS1 spectra were collected in the range of 350–1500 m/z for 250 ms. The 20 most intense precursors with a charge state of 2–5 were selected for fragmentation, and the MS2 spectra were collected in the range of 50–2000 m/z for 100 ms; precursor ions were excluded from reselection for 15 s.

The original MS/MS file data were submitted to the ProteinPilot Software v4.5 for data analysis. For protein identification, the Paragon algorithm2, which was integrated into ProteinPilot, was employed against the Solanum tuberosum transcriptome database (107,739 items) for database searching. The parameters were set as follows: the instrument was TripleTOF 5600 (AB SCIEX Company), iTRAQ quantification, cysteine-modified with iodoacetamide; biological modifications were selected as ID focus, trypsin digestion, the Quantitate, Bias Correction and Background Correction was checked for protein quantification and normalization. For the false discovery rate (FDR) calculation, an automatic decoy database search strategy was employed to estimate the FDR using the PSPEP (Proteomics System Performance Evaluation Pipeline Software, integrated in the ProteinPilot Software) (ProteinPilot™ Software 5.0) algorithm. Only proteins with at least one unique peptide and an unused value of more than 1.3 were considered for further analysis.

### 2.4. Bioinformatic Analysis of the HPLC-MS/MS Data

The average fold-change after normalization, induced by treatment relative to the control, was defined as the fold change for the biological or technical replicate experiments. The statistical significance of the difference in protein expression levels between the samples was determined using a two-tailed Student’s *t*-test. The minimum *p*-value from the replicate experiments was used as the final *p*-value. Data are presented as mean ± SD. For the protein abundance ratios measured using iTRAQ, a 1.5-fold change and a *p*-value less than 0.05 were considered the threshold for identifying significant changes. Hierarchical clustering was applied to differentially abundant proteins (DAPs) using the Euclidean distance analysis, with the linkage type set to “average”. All the expression values were log-transformed with base 2 before calculating the distances. Hierarchical clustering was performed using the Genesis software (Genesis).

### 2.5. Statistical Analysis

All statistical analyses and correlation analyses in this study were realized through R (4.0.1). The correlation analysis was performed using the Pearson correlation coefficient, and the statistical test was conducted using the Student’s *t*-test (*p*-value < 0.05). All the visualization was carried out using pheatmap (1.0.12) to realize heatmaps.

## 3. Results

### 3.1. Statistics of Proteome Characteristics Based on iTRAQ

**Figure 1:**
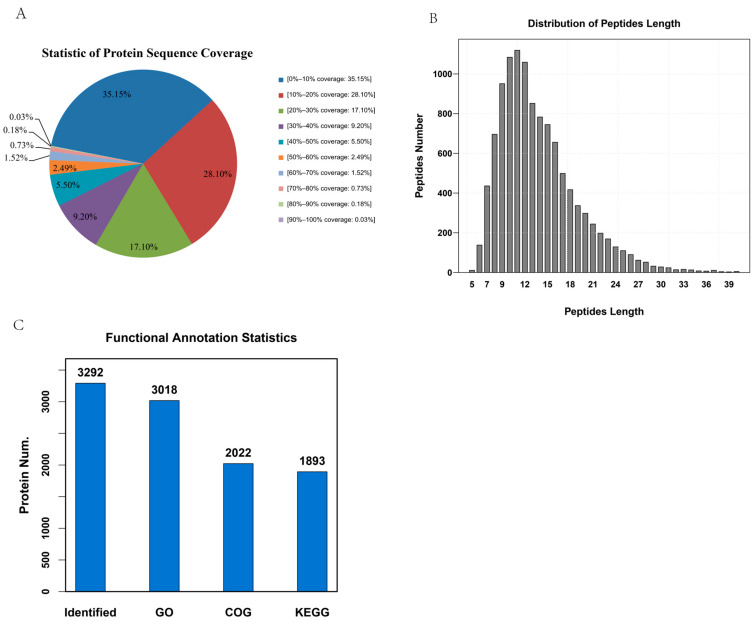
Potato proteome iTRAQ quantitative analysis feature statistics. (**A**) Quantitative coverage of the proteome. Colors represent the different ranges of sequence coverage, and percentages show the proportion of proteins within each coverage interval. (**B**) Average peptide chain length. (**C**) Annotation information statistics of the three major proteome databases.

To determine the changes in the potato proteome in response to low-temperature stress, we employed a protein quantification method based on iTRAQ. The distribution of protein coverage indicated that the majority of identified proteins exhibited relatively low sequence coverage. Specifically, 62.4% of proteins had coverage below 20%, and only 14.7% showed coverage greater than 40% (Figure 1A). This skew toward lower coverage suggests a degree of data loss that is inherent to the iTRAQ method, likely due to limitations in peptide detection and identification efficiency. The process revealed a large number of peptide chains, with the length of these peptide chains mainly ranging from 8 to 17 aa (Figure 1B). Of the 3292 proteins detected in the four tuber tissues across the four different growth environments, 3018 were successfully annotated in the Gene Ontology (GO) database, 1893 in the KEGG database, and 2022 in the Clusters of Orthologous Groups (COG) database (COG) (Figure 1C).

### 3.2. Protein Classification of the Potato Proteome

**Figure 2:**
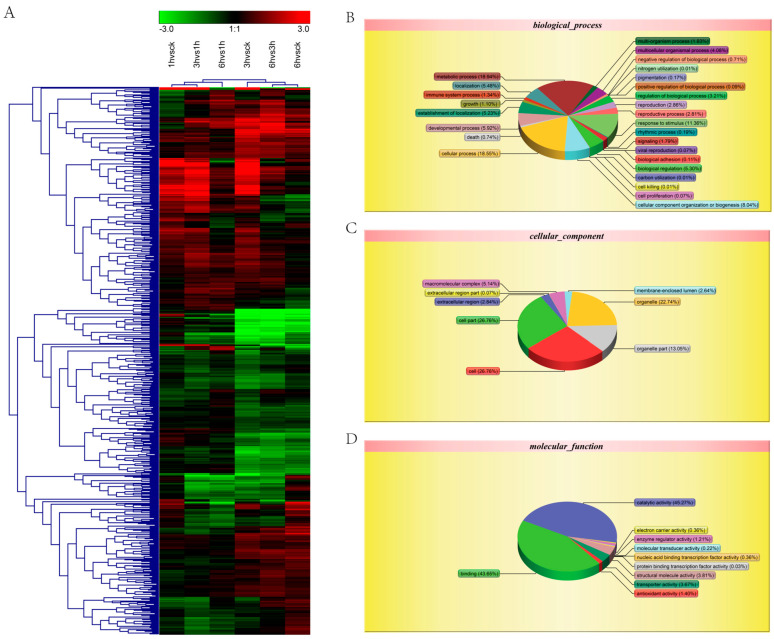
Potato proteome global expression changes and protein classification. (**A**) Potato proteome global expression changes. (**B**–**D**) GO function enrichment analysis of biological process proteins (**B**), cellular component proteins (**C**), and molecular function proteins (**D**).

To further evaluate the function of the potato proteome, all the detected proteins were annotated using the GO database. Among the 3292 identified proteins, 3018 were successfully assigned GO terms, covering three main GO categories: biological process, cellular component, and molecular function. A total of 45 GO terms were associated with these proteins (Figure 2A), which may be associated with the low-temperature tolerance of potato. In the biological process category (Figure 2B), 18.94% of the proteins were related to the metabolic processes, 11.36% to responses to stimuli, 8.04% to cellular component organization or biogenesis, and 5.48% to localization, with the remaining proteins distributed across 23 other terms. In the cellular component category, the proteins were annotated to terms such as cell part, organelle, macromolecular complex, and extracellular region (Figure 2C). In the molecular function category, the annotated terms included binding, catalytic activity, electron carrier activity, enzyme regulator activity, molecular transducer activity, nucleic acid binding transcription factor activity, protein binding transcription factor activity, structural molecule activity, transporter activity, and antioxidant activity (Figure 2D).

### 3.3. Difference Analysis and Functional Analysis of GO and KEGG of DAPs

**Figure 3:**
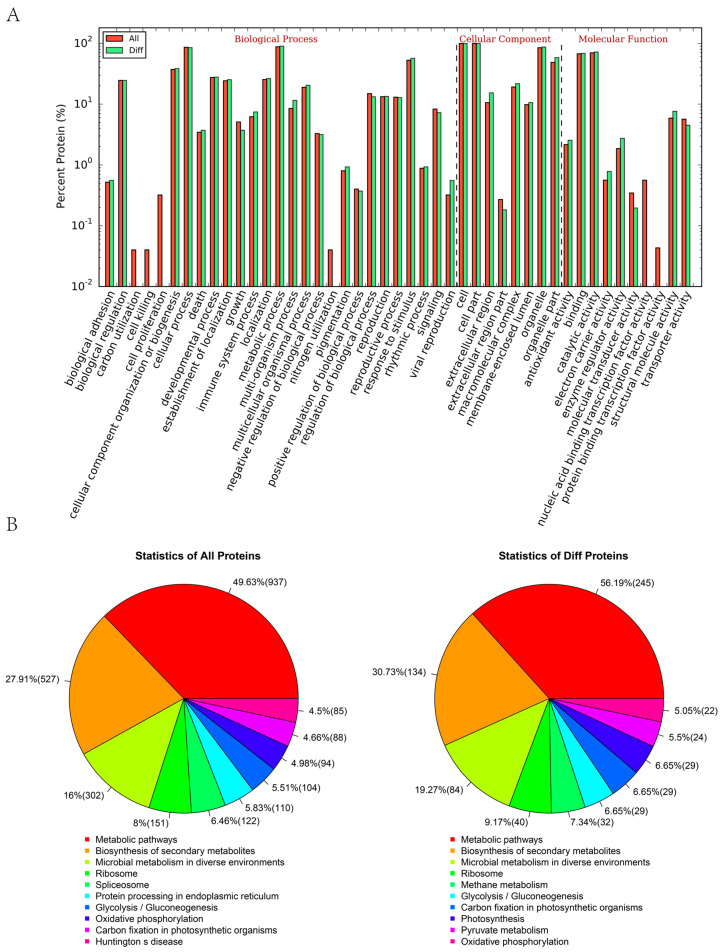
Function analysis of the differential protein in the potato proteome. (**A**) GO function en-richment analysis of DAPs. (**B**) KEGG pathway enrichment analysis of DAPs.

To determine the protein expression changes of potato during the response to low-temperature stress, we conducted a comparative analysis of the protein differences across all groups. In comparison with NT, 125, 250, and 380 DAPs were identified in the L1 (low-temperature growth for 1 h), L3 (low-temperature growth for 3 h), and L6 (low-temperature growth for 6 h) tubers, respectively. To identify the enriched GO functional group of the DAPs, we performed a GO analysis on all the DAPs and found that they were enriched in 39 different GO terms (Figure 3A). The proportions of the enriched DAPs were categorized into three main GO categories. In the biological process category, significantly enriched GO terms included biological adhesion, biological regulation, and developmental processes. For the cellular component category, significant enrichment was observed in cell- and organelle-related terms. In the molecular function category, transporter activity, structural molecule activity, and enzyme regulator activity were notably enriched. To further explore the biological functions of the DAPs, we conducted an enrichment analysis using the KEGG database. The analysis revealed that the DAPs were enriched in 10 pathways, including metabolic pathways, the biosynthesis of secondary metabolites, microbial metabolism in diverse environments, ribosome, methane metabolism, glycolysis/gluconeogenesis, carbon fixation in photosynthetic organism, photosynthesis, pyruvate metabolism, and oxidative phosphorylation (Figure 3B).

### 3.4. Proteome and Transcriptome Association Analysis

**Figure 4:**
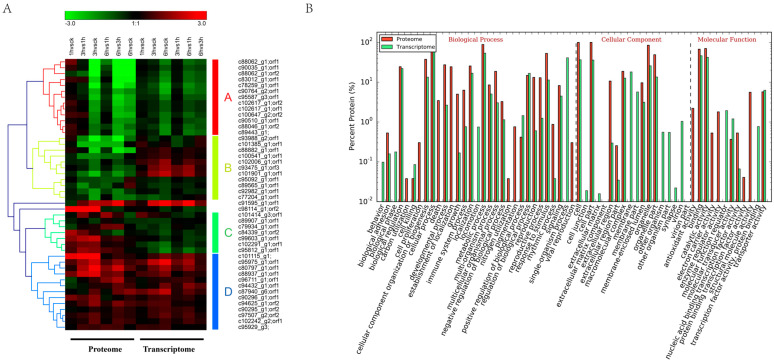
Association analysis of potato proteome and transcriptome. (**A**) Four modules identified through association analysis, including the simultaneous up-regulation of mRNA and protein, the simultaneous down-regulation of mRNA and protein, the down-regulation of mRNA and up-regulation of protein, and the up-regulation of mRNA and down-regulation of protein. (**B**) GO function enrichment analysis of these modular genes.

To gain a comprehensive understanding of gene expression, we conducted a joint analysis of the potato proteome and transcriptome data under low-temperature stress. Four key gene modules were identified: module A, module B, module C, and module D (Figure 4A), comprising a total of 49 genes. Module A contained 13 genes, all of which showed down-regulation at both the protein and mRNA levels in all the stages. Module B contained 11 genes, exhibiting up-regulated expression in transcribed water products and down-regulated expression in protein water products. Module C comprised seven genes that displayed an opposite expression pattern to module B. Module D contained 13 genes, all of which showed consistent up-regulation at both the transcriptional and protein levels across all stages. These findings suggest that those key genes may be closely related to potato cold tolerance. At the same time, we analyzed the pathways that the transcriptome and proteome genes were enriched in, identifying biological regulation, membrane-enclosed lumen, and binding pathways (Figure 4B). These pathways correspond to the classification of the biological process, cellular component, and molecular function, respectively. Additionally, only 13 genes (Module D) exhibited consistent expression trends at both the transcript and protein levels, indicating a weak correlation between the transcriptome and proteome.

### 3.5. Critical Pathways in the Different Cold Stress Stages of Potato

**Figure 5:**
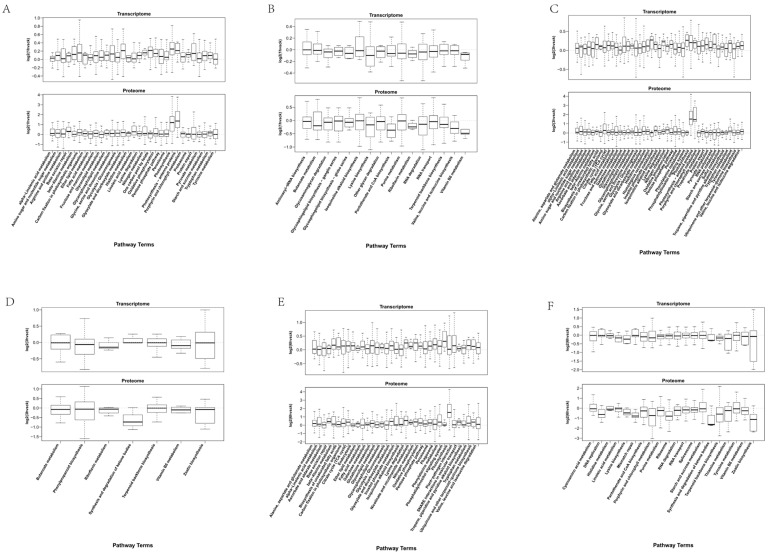
KEGG function enrichment analysis of the genes involved in potato mRNA and protein at different cold stress stages. (**A**) L1 up-regulated genes. (**B**) L1 down-regulated genes. (**C**) L3 up-regulated genes. (**D**) L3 down-regulated genes. (**E**) L6 up-regulated genes. (**F**) L6 down-regulated genes.

To further analyze the dynamic changes in the transcriptome and proteome at each stage of cold stress, we analyzed the genes involved across all the stages. After 1 h of low-temperature growth, 16 were pathways involved in genes down-regulated by both the transcriptome and proteome, including aminoacyl-tRNA biosynthesis, butanoate metabolism, and glycosaminoglycan degradation (Figure 5B). The up-regulated genes were enriched in 32 KEGG pathways, such as alpha-linolenic acid metabolism, amino sugar and nucleotide sugar metabolism, arginine and proline metabolism, and base excision repair, etc. (Figure 5A). After 3 h of low-temperature growth, the co-down-regulated genes identified in both the transcriptome and proteome were enriched in seven pathways, including butanoate metabolism, phenylpropanoid biosynthesis, riboflavin metabolism, synthesis and degradation of ketone bodies, terpenoid backbone biosynthesis, vitamin B6 metabolism, and zeatin biosynthesis (Figure 5D). The up-regulated genes enriched in 43 KEGG pathways, which were alanine, aspartate and glutamate metabolism, alpha-linolenic acid metabolism, etc. (Figure 5C). After 6 h of low-temperature growth, the down-regulated genes were enriched in 20 pathways, including cyanoamino acid metabolism, DNA replication, and histidine metabolism, etc. (Figure 5F). The commonly up-regulated genes were enriched in 35 KEGG pathways, which were alanine, aspartate and glutamate metabolism, and alpha-linolenic acid metabolism, etc. (Figure 5E). The results revealed the molecular pathways that were specifically activated at each stage, providing insight into the mechanisms underlying cold tolerance in potato.

### 3.6. Expression Profile of Stress Response DAPs Involved in Cold Stress

**Figure 6:**
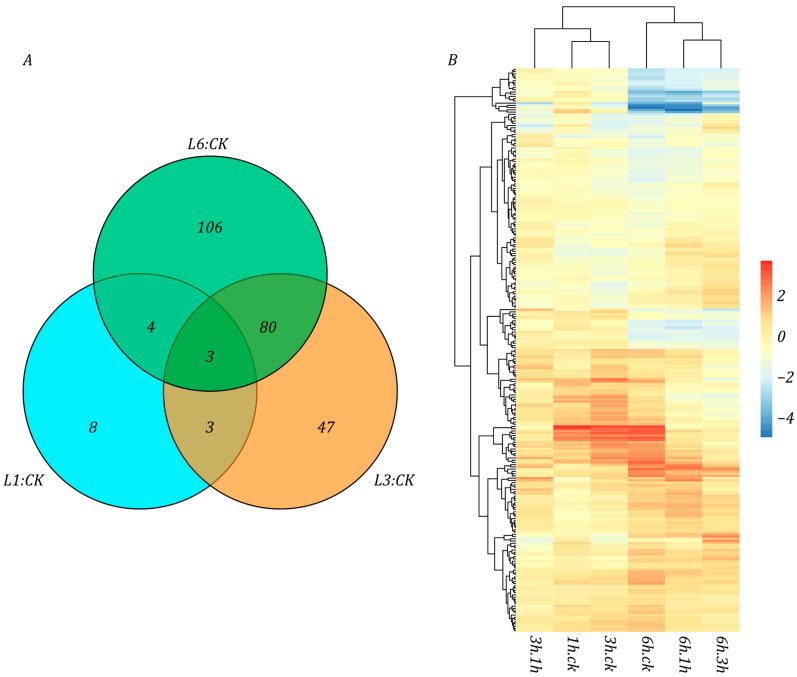
The change in DAPs related to the pressure response of potato under low-temperature growth conditions. (**A**) Venn diagram of DAPs in the three low-temperature stages. (**B**) Heatmap of the change multiple of the pressure response-related DAPs at all stages.

Low temperature alters plant homeostasis, including gene expression. In general, when plants are exposed to low-temperature stress, their metabolic activity decreases significantly, while stress-related genes are activated to maintain the normal physiological activities of cells. Compared with potato grown under normal conditions, 18, 133, and 193 DAPs related to stress response were found at the L1, L3, and L6 stages, respectively (Figure 6A). Among these DAPs, 83 were shared by the L3 and L6 stages, while L1 and L6, and L1 and L3 have 6 and 7 common DAPs, respectively. Most DAPs were activated and expressed during the L3 and L6 stages, with the L6 stage maintaining a high expression level. In contrast, the majority of those DAPs did not change significantly at the L1 stage (Figure 6B). This indicates that the potato activates a series of stress response-related genes after growing under low temperature conditions for 3 h, with the up-regulated expression of these genes ensuring its physiological activities under cold conditions.

### 3.7. Expression Profile of Secondary Metabolites Related to DAPs Involved in the Cold Response

**Figure 7:**
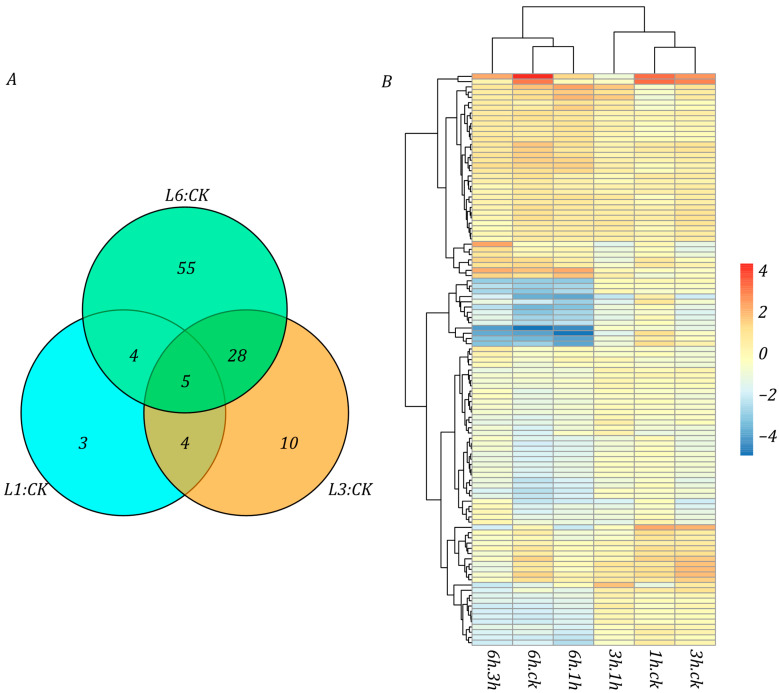
Changes of DAPs related to secondary metabolites of potato under low temperature growth conditions (**A**) The Venn diagram of DAPs in the three low-temperature stages. (**B**) Heatmap of the multiple changes of DAPs related to secondary metabolites at all stages.

Secondary metabolites play a major role in the plant adaptation to environmental stress. Secondary metabolites are not directly involved in the normal growth, development, or reproduction of the organism; conversely, they generally mediate ecological interactions, which may result in a selective advantage for the organism by increasing its survivability or fecundity. However, the relationship between low-temperature growth in potato and the regulation of secondary metabolites remains largely unclear. The proteomic analysis revealed 16, 47, and 92 DAPs associated with secondary metabolism at the L1, L3, and L6 stages, respectively, compared with the control group; however, the changes at L1 were relatively modest and may not be statistically significant (Figure 7A). Further analysis showed that, unlike stress-responsive genes, proteins related to secondary metabolites did not show significant differences at the L1 stage, and most of the related proteins showed an upward expression trend at the L1 stage. At the L3 and L6 stages, the content of many proteins showed a downward trend (Figure 7B). Particularly, there were 18 general secondary metabolite-related genes showing down-regulated expression in the L6 stage. This indicated that the secondary metabolic pathways may be activated in the primary stage of low-temperature response, and as the exposure to cold extends, the activity of these pathways is inhibited to a certain extent.

### 3.8. Dynamic Changes of Immune System- and Carbohydrate Metabolism-Related DAPs

**Figure 8:**
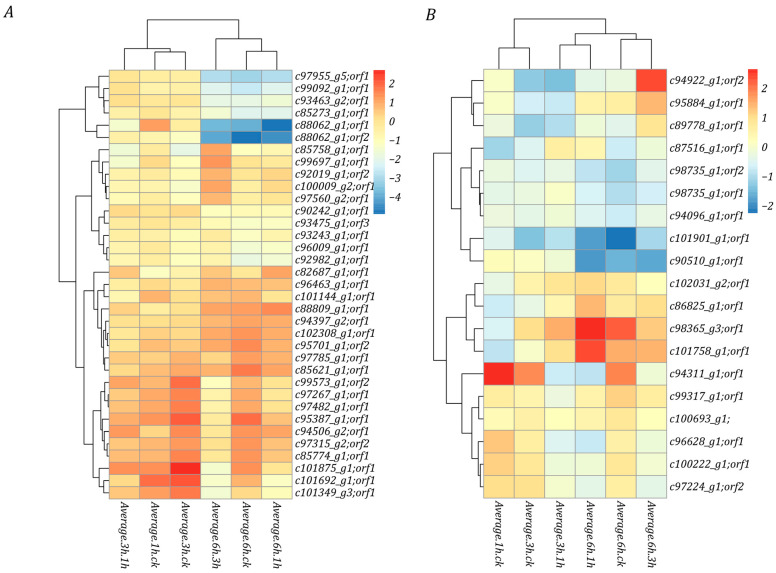
Compared with the control group, the potato DAPs’ abundance change folds under the three stages of L1, L3, and L6. (**A**) Heatmap of the multiple changes in DAPs related to the plant immune system. (**B**) Heatmap of the multiple changes in DAPs related to starch and sucrose metabolism.

The proteomic analysis revealed 36 DAPs that were closely associated with immune system function. Compared with the NT, 6, 17, and 25 DAPs were found at the L1, L3, and L6 stages, respectively (Figure 8A). Further assessment found that, while most of DAPs did not change significantly, only a small number of them showed up-regulated expression at the L1 stage. As the duration of cold exposure increased, the immune system-related DAPs that were activated subsequently also gradually increased. At the L6 stage, most DAPs showed significant up-regulation changes, but some DAPs showed significant down-regulation changes compared with the L3 stage, suggesting that prolonged low-temperature treatment elicits a response from the immune system.

At the same time, significant changes were observed in proteins involved in starch and sucrose metabolism. A total of 20 DAPs related to these metabolisms has been identified. Compared with NT, 4, 11, and 18 DAPs were found at the L1, L3, and L6 stages, respectively (Figure 8B). Overall, the changes in these proteins showed a downward trend. With prolonged exposure to low temperatures, the synthesis of starch in potato was inhibited and the decomposition became more active. This demonstrated that potato dealt with continuous low-temperature stress by breaking down stored carbohydrates.

## 4. Discussion

Low temperature significantly affects potato cultivation and production. Analyzing the molecular mechanism of cold-tolerant strains to low-temperature tolerance is crucial in potato genetic breeding efforts [30,31,32,33]. Proteomics analysis offers extensive insight into the individual proteins involved in specific biological responses. Recently, the genome-wide detection of stress-responsive genes through omics analysis has been increasingly applied to various crops under different stress conditions [34,35]. Studies have examined the gene expression of rice, sesame, cotton, corn, and other species kinetics under low-temperature conditions [36,37,38,39,40]. However, omics research on the cold response and tolerance of potato remains limited.

In this study, iTRAQ-based proteomics data revealed significant molecular pathway changes in potatoes during different stages of cold stress (NT, L1, L3, L6). Our analysis showed that potato activates distinct molecular pathways at each stage to respond to low-temperature stress. Interestingly, the correlation between the proteome and transcriptome data was weak, with gene expression at the mRNA level often not aligning with the corresponding protein levels, as seen in previous studies [41,42,43]. This discrepancy may stem from current technical limitations in transcriptome and proteome analyses, which hinder the accurate detection of gene expression levels across these two omics platforms.

To address this, we focused on genes and molecular pathways that exhibited coordinated changes at both the transcriptomic and proteomic levels. The results revealed that, compared with the NT group, 32 pathways were jointly up-regulated at the L1 stage. Among these, 16 pathways contained genes that were commonly up-regulated in both the transcriptome and proteome. Among them, up-regulated genes were enriched in pathways such as alpha-linolenic acid metabolism, amino sugar and nucleotide sugar metabolism, arginine and proline metabolism, and base excision repair. Alpha-linolenic acid metabolism, which is part of the basic defense network against abiotic and biotic stress in *Arabidopsis* [44], likely plays a similar role in potato under cold stress.

Base excision repair (BER), a critical genome defense pathway, repairs non-voluminous DNA lesions induced by endogenous or exogenous genotoxic agents [45,46]. This complex process was initiated by the excision of the damaged base, and proceeds through a sequence of reactions that generate various DNA intermediates and culminates with the restoration of the original DNA structure [47]. Research has shown that the activity of BER enzymes significantly increases under biotic and abiotic stress [48], suggesting that the BER pathway may also be important in the cold stress response of potato. Cold exposure may induce oxidative stress in potato, and the activation of the BER pathway is likely critical in repairing oxidative damage to mitochondrial DNA. At the same time, we also found a number of molecular pathways related to down-regulated genes, such as aminoacyl-tRNA biosynthesis, butanoate metabolism, and glycosaminoglycan degradation. These pathways are critical for various cellular functions and their down-regulation under cold stress may reflect the reorganization of cellular priorities in response to the cold environment. For example, aminoacyl-tRNA biosynthesis is essential for protein synthesis [49], and its down-regulation could indicate a reduction in protein translation or the shift toward more energy-efficient processes during cold-induced stress. Similarly, the down-regulation of glycosaminoglycan degradation, which is involved in the breakdown of complex carbohydrates [50], suggests that the plant may be prioritizing structural integrity and the protection of cellular membranes under stress conditions. These findings highlight how the plant adapts at the molecular level to mitigate the effects of oxidative damage and maintain cellular function under cold stress.

Gamma-aminobutyric acid (GABA) plays a dual role as both a signaling molecule and a metabolite involved in energy regulation during plant growth and development. It is a four-carbon non-protein amino acid commonly found in various organisms in a free state [51]. Under environmental stress, plants typically exhibit rapid and substantial accumulation of GABA, which enhances cellular responses and stress adaptability [52,53]. In this study, however, we observed a slight reduction in the mRNA expression of GABA-related genes at the L1 stage, accompanied by a significant decrease in protein abundance. This discrepancy suggests that GABA may not be actively involved in the early response to cold stress in potato, or that the stress conditions applied were insufficient to fully trigger the GABA-associated pathways. In contrast, molecular responses at the L3 stage differed markedly, with 43 pathways showing up-regulation and common genes enriched across 7 pathways. These findings indicate a broader and more robust activation of molecular pathways as cold stress progressed. Notably, the key up-regulated pathways at L3 included ascorbate and aldarate metabolism, alanine, aspartate and glutamate metabolism, alpha-linolenic acid metabolism, and other energy-related metabolic processes.

The tolerance of plants to high and low temperatures is linked to their ascorbate content and related enzyme activities. Studies have shown that potato leaves significantly increase their ascorbate content and the expression of related metabolic enzymes when exposed to low temperatures [54]. The level of dehydroascorbate, an oxidation product of ascorbate, also increased significantly, highlighting the fundamental role of ascorbate in the temperature stress resistance of potato. Under stress, the activity of the antioxidant system is enhanced to remove active oxygen and resist environmental damage [55,56]. Airaki et al. demonstrated that pepper plants acclimated to cold by adjusting their antioxidant metabolism, including significant increases in ascorbate levels [57], suggesting that ascorbic acid plays a vital role in cold resistance during short-term cold exposure. Our research indicated that ascorbate may be crucial in the response of potato to low temperature stress for 3 h. However, no activation of ascorbate-related pathways was found at 1 h of treatment. At the L3 stage, the down-regulated genes were enriched in butanoate metabolism, phenylpropanoid biosynthesis, and riboflavin metabolism, etc., most of which were also down-regulated at the L1 stage. At the L6 stage, the up-regulated genes were enriched in ascorbate and aldarate metabolism, alanine, aspartate and glutamate metabolism, and base excision repair, etc. These pathways have already been activated at the L1 and L3 stages and continue to be activated at the L6 stage. The number of down-regulated pathways increased to 20 at the L3 stage, including cyanoamino acid metabolism, DNA replication, histidine metabolism, lysine biosynthesis, and mismatch repair, etc. These findings showed that 6 h of cold stress significantly affects the amino acid synthesis and normal DNA synthesis in potato, indicating a substantial impact on growth and development.

This research offers valuable insights into the candidate genes and proteins involved in the cold stress response in potato. To our knowledge, this is the first proteome analysis addressing the cold stress response in potato, offering new avenues for understanding and improving cold tolerance in this important crop.

## Data Availability

All data and materials are presented in the main paper.
